# Generalist species exhibit more genetic structure in comparison to a habitat specialist: Evidence from a phylogeographic study of two freshwater crabs (Decapoda: Potamonautidae: *Potamonautes*), implications for habitat conservation

**DOI:** 10.1002/ece3.70285

**Published:** 2024-09-13

**Authors:** Petrus C. J. Grobler, Savel R. Daniels

**Affiliations:** ^1^ Evolutionary Genomics Group, Department of Botany and Zoology Stellenbosch University Matieland South Africa

**Keywords:** aquatic invertebrates, biogeography, dispersal, lentic, lotic, speciation

## Abstract

During the present study, the phylogeography of the wetland specialist, freshwater crab, *Potamonautes flavusjo*, was investigated and compared to that of the ubiquitous, generalist, *P. sidneyi*, using DNA sequence data from the mitochondrial cytochrome oxidase subunit one (COI). We inferred the evolutionary history of each species and compared their population‐level genetic structure by constructing haplotype networks and using an analysis of molecular variation. Additionally, we explored the evolutionary relationship between southern African lentic and lotic freshwater crab species by examining the usefulness of carapace attributes in relation to genetic indices and a species' assumed dispersal capacity. In the lentic species, *P. flavusjo*, a single interconnected haplocluster characterized by shared haplotypes was observed, suggesting marked maternal dispersal, a result corroborated by the low *F*_ST_ values. In contrast, for the lotic species, *P. sidneyi* two distinct haploclusters and marked genetic differentiation was observed indicating the absence of maternal dispersal, a result corroborated by the high *F*_ST_ values. The phylogenetic relationship in *P. sidneyi* was further investigated using a maximum likelihood and Bayesian inference analyses with the addition of sequence data from the mitochondrial 16S rRNA locus to estimate divergence times. Three species delimitation methods (ASAP, PTP, and bGMYC) were used to explore the presence of distinct lineages in *P. sidneyi*. The phylogenetic results indicated that within *P. sidneyi* two clades were present, while divergence time estimates suggest cladogenesis during the middle to late Pleistocene. The species delimitation methods used showed moderate congruence, however all oversplit the number of putative lineages. Our results indicated that *P. sidneyi* is a species complex comprised of two cryptic lineages, occurring in sympatry contemporarily possibly alluding to secondary contact. Carapace height was found to have no discernible influence on the genetic indices and presumed dispersal capabilities of mainland southern African freshwater crab species. The importance of our results are discussed in terms of conservation of freshwater habitats.

## Graphical abstract

The study investigated the phylogeography of two freshwater crab species, *Potamonautes flavusjo* and *P. sidneyi*, comparing their evolutionary history and population genetic structure using DNA sequencing. While *P. flavusjo* showed evidence of marked maternal dispersal and low genetic differentiation, *P. sidneyi* exhibited significant genetic divergence and absence of maternal dispersal. Additionally, the study explored the relationship between carapace attributes and genetic indices, finding no significant influence on dispersal capabilities. These findings have implications for the conservation of freshwater habitats.

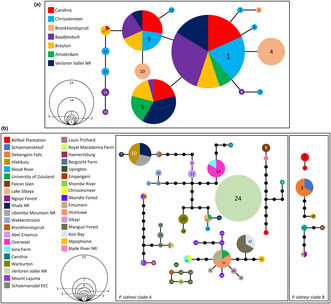

## 1 INTRODUCTION

Inland freshwater environments, such as, rivers, streams, wetlands and ephemeral pans harbor a unique guild of invertebrates whose habitat are frequently subjected to fragmentation induced by ancient and recent abiotic events, such as geological and climatic ameliorations (Emerson, 2002; Flagstad et al., 2001; Hewitt, 2001). These episodic habitat fragmentation events, depending on the duration of spatiotemporal isolation and the absence of conspecific dispersal between fragments will leave its genetic signature on the phylogeography structure of freshwater species (Hughes et al., 2004; Marske et al., 2020; Zamudio et al., 2016). In both lentic (standing) and lotic (running) freshwater habitats, invertebrate taxa are seasonally exposed to fluctuations in precipitation resulting in habitat inundation and expansion, followed by periods of drought resulting in habitat contraction, with mesic periods such as floods likely facilitating dispersal events. Consequently, both lentic and lotic taxa exhibit a diverse repertoire of adaptive strategies that allow freshwater taxa to survive in their habitats (Buffagni et al., 2010; France & Duffy, 2006; Lobera et al., 2019; Starzomski & Srivastava, 2007). The oscillatory nature of climate in conjunction with geomorphological processes together with the organism's dispersal capability will be reflected in the phylogeographic structure of the species (Bentley et al., 2014; Couvreur et al., 2021; Goudie, 2005; Zhang et al., 2020).

Lentic habitats, exemplified by the Great East African Lakes, exhibit both ancient and recent origins (Daniels et al., 2015; Renaut & Owen, 2023; Salzburger et al., 2014). The rifting process in the East African system was initiated approximately 30 million years ago during the Oligocene epoch (Burke, 1996; Goudie, 2005). However, Lake Tanganyika's formation began between 9 and 12 million years ago, while in neighboring Lake Malawi, the formation was initiated during the late Miocene around 8.6 million years ago (Cohen et al., 2016; Contreras et al., 2000; Daniels et al., 2015; Delvaux, 1995; Renaut & Owen, 2023). Fluctuations in lake levels created a multitude of ecological niches that are thought to be one of the major driving forces for the spectacular endemism and species diversity observed in the Great East African Lakes (Ronco et al., 2020; Salzburger, 2018; Salzburger et al., 2014; Seehausen, 2015; Sturmbauer et al., 2001). Lotic systems in Africa are ancient and dynamic ecosystems that have undergone considerable river capture as a result of orogenesis, frequently shifting direction of flow and severing hydrological connection (Goudie, 2005; Stankiewicz & de Wit, 2006). For example, in southern Africa, the upper and lower segments of the Zambezi River were previously distinct systems, with the upper segment linked to the Limpopo and the middle Zambezi connected to the Shire systems (Goudie, 2005; Moore et al., 2009; Moore & Larkin, 2001). These connections are believed to have persisted until as recently as the Pliocene or mid‐Pleistocene (Thomas & Shaw, 1991), facilitating faunal exchanges (Lowe‐McConnell, 1993). The plasticity of river systems may act as a catalyst for the formation of faunal boundaries laying the foundation for subsequent speciation (Brown & Swan, 2010; Carrara et al., 2012; Daniels et al., 2015; Oberdorff et al., 2019; Zhang et al., 2020). Investigating the evolutionary history of obligatory freshwater invertebrates could provide valuable insights into how climatic ameliorations drove diversification for invertebrate taxa inhabiting both lentic and lotic environments (Bentley et al., 2014; Daniels et al., 2015; Sands et al., 2022).

Invertebrates form key components of the aquatic food web and represent a suitable group with which to test the impact of habitat fragmentation on a species phylogeographic structure. Freshwater crabs are the largest macroinvertebrates in the Afrotropical region and central to ecological processes (Butler & Marshall, 1996; Cumberlidge et al., 2009; Purves et al., 1994). They are present in both lentic and lotic habitats and possess varying dispersal capabilities, produce large lecithotrophic eggs, lack larval dispersal and exhibit extensive maternal care (Daniels et al., 2006, 2015; Liu & Li, 2000). Adult freshwater crabs can be amphibious to a varying degree depending on their ecophysiology, resulting in varying levels of phylogeographic differentiation (Barbaresi et al., 1997; Daniels et al., 2003, 2006, 2020; Wolcott, 1988; S. R. Daniels, personal communication). For example, semi‐terrestrial species such as *Maritimonautes calcaratus* and *Potamonautes lividus* are thought to have a better dispersal capability than lotic dwelling species such as *P. sidneyi* or *P. perlatus* (Daniels et al., 2003, 2006, 2020, 2023; Gouws et al., 2015). This suggests that semi‐terrestrial, lentic‐dwelling taxa could possibly have low genetic variation, while lotic taxa might be characterized by marked genetic differentiation and the presence of cryptic lineages. In freshwater crabs, the species carapace dimensions, (carapace length and height), have proven to be valuable indices for assumed habitat preferences (Cumberlidge, 1999). For example, a wide and relatively flat carapace is associated with a fully aquatic existence in large streams, major rivers and lakes (Cumberlidge, 1999), whereas a moderately high or exceptionally high carapace is associated with modifications of the branchial chambers for aerial respiration and is typical of species with a semiterrestrial or terrestrial lifestyle (Cumberlidge, 1999). Therefore, the carapace height (CH) over carapace length (CL) can be used to assess the dispersal capability of freshwater crab species. Hence, a species with a higher height coefficient (CH/CL) may suggest a more terrestrial lifestyle, potentially indicating enhanced dispersal capabilities, and genetic connectivity. However, the relationship between carapace morphology and genetic variability has to date not been examined in freshwater crabs.

*Potamonautes flavusjo* a semi‐terrestrial burrowing freshwater crab that occurs in wetland areas (lentic habitat) was originally described from the Highveld of the Mpumalanga province and recently discovered in the Gauteng province of South Africa (Daniels et al., 2014; P. C.J. Grobler & S. R. Daniels, personal observation). The Highveld is a plateau at an altitude above 1500 m above sea level (a.s.l.), but lower than 2100 m a.s.l. (Daniels et al., 2014, 2016). These wetland habitats are highly fragmented (Grobler pers. obs.). The extensive burrowing behavior and bioturbation of the freshwater crab species potentially function as a mechanism for ecosystem engineering, facilitating the aeration and mixing of peat soil commonly found in wetland areas (Grobler pers. obs.). Carapace indices (CH/CL = 0.65) suggest *P. flavusjo* potentially has a high dispersal capability and should exhibit genetic invariance, however, the species' phylogeographic structure remains unknown. Conversely, *P. sidneyi* is the most ubiquitous of all South African freshwater crabs and is present in seven provinces of the country, excluding only the Eastern and Western Cape provinces (Barnard, 1950; Daniels et al., 2023; Peer et al., 2017; Stewart & Cook, 1998). *Potamonautes sidneyi* inhabits a diverse array of aquatic environments, including rivers (lotic), streams, wetlands, and farm dams, occurring from sea level to elevations exceeding 1000 m a.s.l. where it frequently occurs in sympatry with *P. flavusjo* on the Highveld (Daniels et al., 2014, 2023; P. C. J. Grobler & S. R. Daniels, personal observation). The generalist nature of *P. sidneyi* enables the species to inhabit various ecological niches within these freshwater environments (Barnard, 1950; Cumberlidge & Daniels, 2008; S. R. Daniels, personal observation). The carapace dimensions of *P. sidneyi* suggest that it is primarily associated with riverine habitats (CH/CL = 0.49) (Cumberlidge, 1999; Daniels et al., 2023). Consequently, it is reasonable to hypothesize that *P. sidneyi* should demonstrate a discernible phylogeographic structure across its expansive distribution range (Gouws et al., 2015). Daniels et al. (2023) conducted limited geographic sampling of *P. sidneyi*, however, evidence from the latter study suggests the possible presence of a cryptic lineage in KwaZulu‐Natal. By undertaking extensive geographic sampling of *P. sidneyi* we further explored the presence of a cryptic lineages.

During the present study, a fine‐scale study of both *P. flavusjo* and *P. sidneyi* was conducted to investigate the role of lentic and lotic systems in relation to carapace dimensions and the phylogeographic structure of the respective species. We aim to elucidate the evolutionary history of *P. flavusjo* and to compare it with that of *P. sidneyi*. Furthermore, our objective is to investigate potential disparities in carapace height between lentic and lotic crab populations and evaluate their implications on dispersal capabilities, thereby shaping their genetic structure. We hypothesize (1) that *P. flavusjo* should exhibit limited genetic differentiation, and presumably have high dispersal capability based on its carapace dimensions, while in *P. sidneyi* marked genetic differentiation should be present owing to its presumed low dispersal capability. Furthermore, we explore the relationship between carapace variables and genetic differentiation and hypothesize (2) that marked carapace height corresponds to shallow genetic variation.

## 2 MATERIALS AND METHODS

### 2.1 Sample collection

**FIGURE 1:**
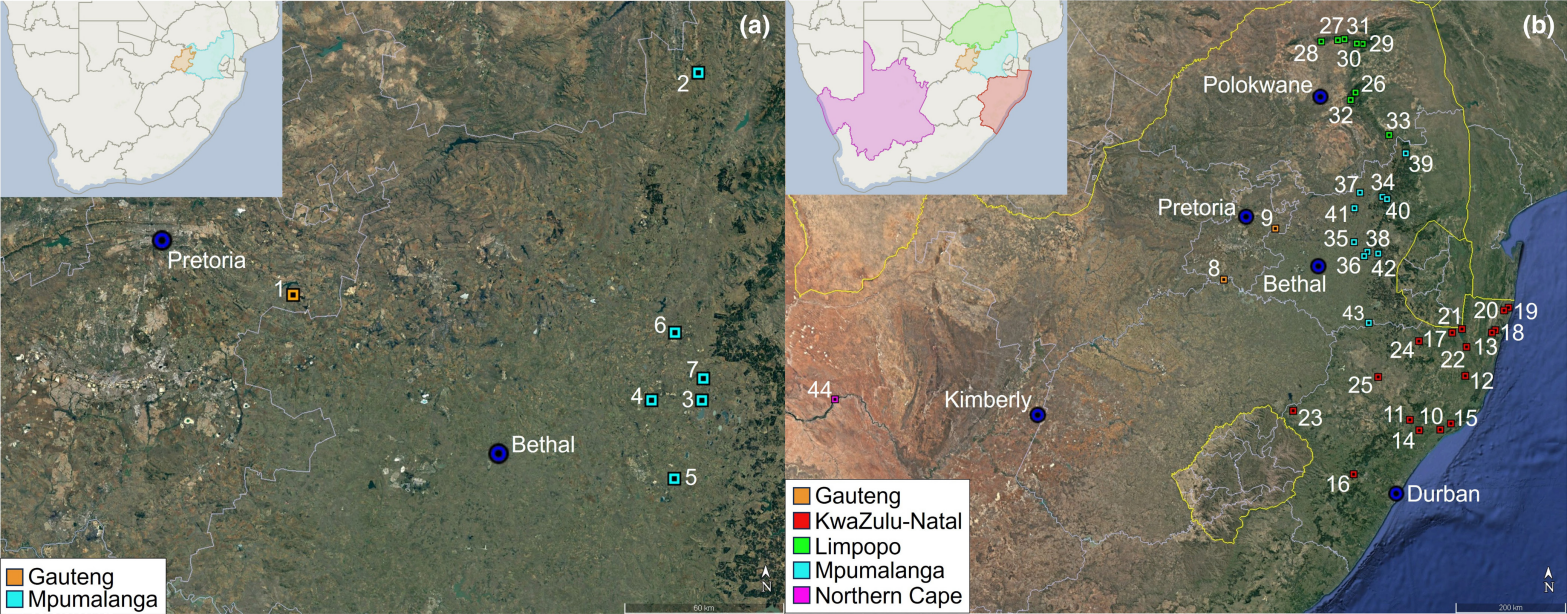
(a) Map showing the seven sample localities from where the freshwater crab *Potamonautes flavusjo* was collected from in the Gauteng and Mpumalanga provinces, South Africa. The sample locality numbers 1–7 on the map correspond to Table 1. (b) Map showing the 37 sample localities from where the freshwater crab species *P. sidneyi* was collected in the Gauteng, Limpopo, KwaZulu‐Natal, Northern Cape and Mpumalanga provinces, South Africa. The sample locality numbers 8–44 correspond to Table 1.

*Potamonautes flavusjo* specimens were hand collected from wetlands in the Gauteng and Mpumalanga provinces of South Africa (Figure 1a; Table 1). Specimens were dug from burrows at the periphery of the wetland with the use of a construction spade. A total of 111 specimens were collected from seven localities and combined with 27 cytochrome oxidase subunit one (COI) sequences from Daniels et al. (2014) to yield a total of 138 sequences (Table 1). *Potamonautes sidneyi* specimens were hand collected or dug up from wetlands where they occurred in sympatry with *P. flavusjo*. In addition, *P. sidneyi* specimens were collected from mountain streams, rivers and dams in Gauteng, Mpumalanga, Limpopo and KwaZulu‐Natal provinces of South Africa (Figure 1b; Table 1). For *P. sidneyi*, a total of 60 specimens were collected from 21 localities during the present study and combined with 55 COI sequences from 16 localities from four previous studies to yield a total of 115 sequences (Table 1) (Daniels et al., 2014, 2019, 2023; Gouws et al., 2015). Locality coordinates were recorded by using a handheld GPS device (Garmin). Recently collected specimens were preserved in 96% ethanol until required for molecular work.

**TABLE 1:** List of localities where specimens of the freshwater crab species *Potamonautes flavusjo* and *P. sidneyi* were collected in South Africa.

Locality #	Locality name	Province	Species	*N*	S	E	Reference study
1	Bronkhorstspruitdam	Gauteng	*P. flavusjo*	23	25°55′50.5″	28°42′42.5″	Present study
2	Verloren Vallei NR	Mpumalanga	*P. flavusjo*	19	25°20′33.3″	30°07′54.6″	Daniels et al. (2014); Present study
3	Chrissiesmeer	Mpumalanga	*P. flavusjo*	25	26°17′55.3″	30°13′41.4″	Daniels et al. (2014); Present study
4	Breyton	Mpumalanga	*P. flavusjo*	17	26°17′00.3″	30°01′56.3″	Present study
5	Amsterdam	Mpumalanga	*P. flavusjo*	8	26°32′37.8″	30°07′11.2″	Present study
6	Carolina	Mpumalanga	*P. flavusjo*	21	26°05′38.1″	30°02′53.8″	Present study
7	Baadjiesbult	Mpumalanga	*P. flavusjo*	25	26°12′39.8″	30°13′31.6″	Daniels et al. (2014); Present study
8	Overwaal	Gauteng	*P. sidneyi*	5	26°42′37.9″	27°51′39.3″	Present study
9	Bronkhorstspruit	Gauteng	*P. sidneyi*	1	25°55′50.5″	28°42′42.5″	Present study
10	Ngoye Forest	KwaZulu‐Natal	*P. sidneyi*	1	28°52′10.2″	31°41′22.9″	Daniels et al. (2019)
11	Nkandla Forest	KwaZulu‐Natal	*P. sidneyi*	4	28°44′10.4″	31°08′05.7″	Daniels et al. (2019, 2023), Gouws et al. (2015)
12	Hluhluwe	KwaZulu‐Natal	*P. sidneyi*	3	28°02′20.0″	32°05′10.0″	Daniels et al. (2019, 2023); Gouws et al. (2015)
13	Lake Sibaya	KwaZulu‐Natal	*P. sidneyi*	3	27°21′50.0″	32°31′35.0″	Gouws et al. (2015)
14	Entumeni	KwaZulu‐Natal	*P. sidneyi*	3	28°53′24.6″	31°18′47.4″	Gouws et al. (2015)
15	Empangeni	KwaZulu‐Natal	*P. sidneyi*	1	28°46′16.2″	31°52′28.1″	Daniels, Stewart, Gouws, et al. (2002)
16	Mpophome	KwaZulu‐Natal	*P. sidneyi*	3	29°34′55.9″	30°11′10.2″	Gouws et al. (2015)
17	University of Zululand	KwaZulu‐Natal	*P. sidneyi*	4	27°23′39.6″	31°48′47.9″	Present study
18	Sibayi	KwaZulu‐Natal	*P. sidneyi*	3	27°19′22.5″	32°35′13.5″	Gouws et al. (2015)
19	Kosi Bay	KwaZulu‐Natal	*P. sidneyi*	2	26° 58′14.7″	32°48′21.4″	Daniels et al. (2023)
20	Manguzi Forest	KwaZulu‐Natal	*P. sidneyi*	4	27°01′03.6″	32°43′32.1″	Daniels et al. (2023)
21	Hlatikulu Forest	KwaZulu‐Natal	*P. sidneyi*	4	27°19′54.8″	31°59′07.2″	Daniels et al. (2023)
22	Ubombo Mountain NR	KwaZulu‐Natal	*P. sidneyi*	2	27°35′59.0″	32°04′54.0″	Present study
23	Khombe River	KwaZulu‐Natal	*P. sidneyi*	1	28°38′46.0″	29°05′18.0″	Present study
24	Ithala NR	KwaZulu‐Natal	*P. sidneyi*	4	27°31′06.1″	31°13′10.3″	Present study
25	Blood River	KwaZulu‐Natal	*P. sidneyi*	2	28°06′19.0″	30°32′30.0″	Present study
26	Debengeni Falls	Limpopo	*P. sidneyi*	5	23°48′52.4″	30°01′51.2″	Present study
27	Schoemansdal EEC	Limpopo	*P. sidneyi*	4	23°00′45.2″	29°43′37.0″	Present study
28	Bergzicht Farm	Limpopo	*P. sidneyi*	1	23°02′49.4″	29°26′52.3″	Present study
29	Royal Macademia Farm	Limpopo	*P. sidneyi*	1	23°03′00.8″	30°08′50.8″	Present study
30	Mount Lajuma	Limpopo	*P. sidneyi*	2	23°02′59.6″	30°02′50.0″	Present study
31	Louis Trichardt	Limpopo	*P. sidneyi*	1	22°59′30.0″	29°50′12.0″	Present study
32	Haenertsburg	Limpopo	*P. sidneyi*	1	23°56′00.0″	29°57′00.0″	Present study
33	Abel Erasmus	Limpopo	*P. sidneyi*	4	24°26′30.3″	30°36′44.2″	Present study
34	Falcon Glen	Mpumalanga	*P. sidneyi*	4	25°23′27.9″	30°30′59.5″	Present study
35	Carolina	Mpumalanga	*P. sidneyi*	1	26°05′38.1″	30°02′53.8″	Present study
36	Chrissiesmeer	Mpumalanga	*P. sidneyi*	1	26°17′55.3″	30°13′41.4″	Daniels et al. (2014)
37	Verloren Vallei NR	Mpumalanga	*P. sidneyi*	14	25°20′33.3″	30°07′54.6″	Daniels et al. (2014)
38	Iona Farm	Mpumalanga	*P. sidneyi*	3	26°13′57.7″	30°16′56.7″	Daniels et al. (2014)
39	Blyde River Canyon NR	Mpumalanga	*P. sidneyi*	1	24°42′36.3″	30°54′11.6″	Daniels et al. (2023)
40	Schoemanskloof	Mpumalanga	*P. sidneyi*	2	25°25′22.1″	30°35′49.0″	Present study
41	Belfast Plantation	Mpumalanga	*P. sidneyi*	5	25°34′59.7″	30°02′35.8″	Present study
42	Warburton	Mpumalanga	*P. sidneyi*	5	26°15′06.6″	30°28′05.1″	Present study
43	Wakkerstroom	Mpumalanga	*P. sidneyi*	5	27°17′56.6″	30°20′20.7″	Present study
44	Upington	Northern Cape	*P. sidneyi*	5	28°27′15.5″	21°14′53.1″	Daniels et al. (2023)

*Note*: The locality numbers (1–44) correspond to the map (Figure 1). *N* represents the number of specimens. Where specimens were incorporated from previous studies, these are listed.

### 2.2 DNA extraction, polymerase chain reaction (PCR) and sequencing

Muscle tissue from walking legs was subjected to DNA extraction using a nucleospin kit (Machery‐Nagel, Duren, Germany), following the manufacturer's protocol. Extracted DNA was stored at −20°C until required for PCR. The primer pair for the single loci is as follows: COI, LCO1‐1490 (5′‐GGTCAACAAATCATAAAGATATTG‐3′) and HCO1‐2198 (5′‐TAAACTTCAGGGTGACCAAAAAATCA‐3′) (Folmer et al., 1994). All samples were sequenced for the COI locus. A geneAmp PCR system Thermocycler (Applied Biosystems, Foster City, CA, USA) was used to conduct the polymerase chain reactions. Standard PCR conditions for amplification and DNA sequencing protocols were followed (Daniels et al., 2015), specifically, a 25 μL reaction was used, consisting of 14.9 μL of deionized water, 3.5 μL of MgCl_2_, 2.5 μL of 10× Mg^2+^ free buffer, forward and reverse primers (0.5 μL each), 0.5 μL of deoxyribonucleotide triphosphate solution, 0.1 μL of Taq polymerase, 2.5 μL of the extracted DNA solution. The PCR conditions for the COI marker were as follows, 94°C (4 min), [94°C (30 s), 42°C (40 s), 72°C (45 s)] for 34 cycles, and a final extension at 72°C (10 min). For *P. sidneyi*, we also sequenced one specimen per locality for 16S rRNA, 16Sa (5′‐ACT TGA TAT ATA ATT AAAGGG CCG‐3′) and 16Sb (5′‐CTG GCG CCG CTC TGAACT CAA ATC‐3′) (Palumbi et al., 1991). Standard PCR conditions for amplification and DNA sequencing protocols were followed (Daniels et al., 2015). Polymerase chain reaction (PCR) products were electrophoresed for 2 h in a 1% agarose gel containing ethidium bromide and a BioFlux gel purification kit was used to clean the products (Bioer Technology Co., Ltd). Sequencing was performed at the Central Analytical Facility of Stellenbosch University.

### 2.3 Phylogeographic analyses sourced from COI data

For both *Potamonautes flavusjo* and *P. sidneyi*, a haplotype network was constructed using TCS 1.21 (Clement et al., 2000), set at 95% confidence. An estimate of population genetic differentiation across all localities (*F*_ST_) was obtained using hierarchical analyses of molecular variance (AMOVA) performed in Arlequin 3.0 (Excoffier et al., 2005) over all *P. flavusjo* sample localities. Similarly, for *P. sidneyi* an AMOVA was conducted over all sample localities. In addition, a second AMOVA was performed for each of the two *P. sidneyi* clades evident from the preliminary analyses. Standard molecular genetic indices were investigated for both species, including the number of polymorphic sites, number of haplotypes, nucleotide and genetic diversity, while Fu's *F*s test (Fu, 1997) was used to examine demographic expansion.

### 2.4 Bayesian skyline analyses for both *P. flavusjo* and *P. sidneyi* using COI data

The Bayesian skyline analysis was conducted under a Bayesian framework which makes use of a probability model to estimate the median heights of species populations through using the Markov Chain Monte Carlo (MCMC) method. A strict molecular clock was implemented through the software BEAST2 v.2.4.8 (Drummond & Rambaut, 2007). The mutation rate for the locus was determined from a fossil‐calibrated phylogeny of Potamonautidae: 2.85% per Myr (SD = 0.005) for the COI locus (Daniels, 2011; Daniels et al., 2015; Daniels & Klaus, 2018). We made use of a multiple coalescent model (Heled & Drummond, 2009) and jModelTest2 v.2.1.6 (Posada, 2008) to define the substitution model parameters for the locus, running fifty million generations for four MCMC chains and performing sampling every 1000 generations to median heights of the populations of *P. flavusjo* and in *P. sidneyi* for clades A and B through time. The skyline plots were generated in Tracer v.1.5 (Rambaut et al., 2014).

### 2.5 Phylogenetic analyses for *P. sidneyi*

Four sister species were selected as outgroups: *P. granularis*, *P. perlatus*, *P. barnardi* and *P. barbari* (Mengel & Daniels, 2024). Maximum likelihood (ML) and Bayesian inference (BI) were used to infer the phylogeny of *P. sidneyi*, ML in RAxML v.7.0.4 (The Exelixis Lab, Heidelberg Institute for Theoretical Studies, Heidelberg, Germany; Stamatakis et al., 2008) and BI in MrBayes v.3.2.2 (http://mrbayes.sourceforge.net/index.php, accessed August 2019; Ronquist et al., 2012). We used IQ‐Tree web server v.1.4.3 (http://iqtree.cibiv.univie.ac.at/; Trifinopoulos et al., 2016) for the ML analyses and tree inference to select for the optimal DNA substitution model and the best‐fit likelihood score, chosen using the Akaike information criterion (AIC) (Akaike, 1973). Bootstrap values of >75% were accepted as sufficient nodal support. Similarly, we used the AIC (Akaike, 1973) to select the optimal DNA substitution model for the BL analyses in jModelTest2 v.2.1.6. (Posada, 2008) on XSEDE through the CIPRES Science Gateway (Miller et al., 2010). Each analysis comprised of four chains for 50 × 10^6^ generations, sampling every 1000 generations using default parameters and selected a random tree for the start of each chain. The burn‐in was included in the command block and set to 20% as discerned in TRACER v.1.6 (Rambaut et al., 2014). After burn‐in, trees were discarded a 50% majority rule consensus tree was generated from retained trees and the posterior probability was provided by the percentage time a node was recovered. A posterior probability (p*P*) of <0.95 was regarded as statistically poorly supported. Uncorrected ‘*p*’‐distances were calculated for the different clades among and within *P. sidneyi* populations for the COI locus in PAUP v.40b10 (Swofford, 2002).

### 2.6 Species delimitation and divergence time estimations from combined COI and 16S rRNA data for *P. sidneyi*

Three species delimitation methods were used during the present study. The newly developed assemble species by automatic partitioning (ASAP) (Puillandre et al., 2021), the Poisson Tree Processes (PTP) (https://species.h‐its.org/ptp/) and the Bayesian implementation of the GMYC model using the R package bGMYC (Reid & Carstens, 2012). The ASAP method was chosen as it uses genetic distances to hierarchically cluster species partitions (https://bioinfo.mnhn.fr/abi/public/asap). The PTP method was chosen due to its ability to delimit species without prior knowledge of population parameters (Zhang et al., 2013). The bGMYC method was chosen because of its robustness towards confounding factors such as mutation rate and unbalanced sampling (Luo et al., 2018). In contrast to ASAP and PTP, the bGMYC method uses a calibrated tree derived from using both COI and 16S rRNA loci. We used the same analytical approach outlined in Daniels et al. (2023).

The divergence time estimate was conducted using a Bayesian framework which uses a probability model to define the molecular sequence divergence of lineages, using the Markov Chain Monte Carlo (MCMC) method to estimate the clade ages. A reduced dataset, representing one sample per locality except for the sympatric localities, where a sample from both clades was used and combined COI and 16S rRNA sequences were used to estimate the divergence time between clades. A strict molecular clock was implemented through the software BEAST2 v.2.4.8 (Drummond & Rambaut, 2007). The mutation rates for each locus were determined from a fossil‐calibrated phylogeny of Potamonautidae: 0.81% per Myr for the 16S rRNA locus (SD = 0.0013), and 2.85% per Myr (SD = 0.0050) for the COI locus (Cumberlidge & Daniels, 2022; Daniels, 2011; Daniels et al., 2015; Daniels & Klaus, 2018). Both loci have rapid substitution rates, making them ideal to detect recent lineage separation, especially in decapods (Daniels, Stewart, & Cook, 2002; Heled & Drummond, 2009; Jesse et al., 2010; Phiri & Daniels, 2014). The maximum clade credibility tree was determined by applying a Yule tree prior in TREEANNOTATOR v.2.4.1 (part of the BEAST package) after 20% of the trees were removed as burn‐in. We made use of a multiple coalescent model (Heled & Drummond, 2009) and jModelTest2 v.2.1.6 (Posada & Crandall, 1998) to define the substitution model parameters for each locus, running 50 million generations for four MCMC chains and performing sampling every 1000 generations to estimate clade ages. The tree was visualized in Tracer v.1.5 to check for convergence (Rambaut et al., 2014).

### 2.7 Morphometric comparison with genetic variation

Carapace height (CH) and carapace length (CL) measurements were obtained from freshwater crab specimens of *P. lividus*, *P. flavusjo*, *P. mariepskoppie*, *P. parvicorpus*, *P. perlatus*, *P. sidneyi*, *P. isimangaliso* and *Maritimonautes calcaratus* using unpublished data (S. R. Daniels, unpublished, Gunkel, unpublished) and data obtained from nine previous studies (Daniels, Stewart, & Cook, 2002; Daniels, Stewart, Gouws, et al., 2002; Gouws et al., 2015; Peer et al., 2015; Phiri & Daniels, 2014; Wood & Daniels, 2016). All measurements were taken in millimeters (mm) using a digital caliper. The mean CH was calculated and divided by the mean CL for each species to obtain the average height coefficient (CH/CL) for each species and species were divided into either lentic or lotic habitats. The mean haplotype and nucleotide diversity for various species, including 138 *P. flavusjo*, 116 *P. mariepskoppie* (Gunkel unpubl.), 37 *P. parvicorpus*, 39 *P. perlatus*, 115 *P. sidneyi*, and 20 *M. calcaratus* specimens, were calculated using COI data from the current and previous studies (Daniels, Stewart, & Cook, 2002; Daniels, Stewart, Gouws, et al., 2002; Gouws et al., 2015; Phiri & Daniels, 2014; Wood & Daniels, 2016). In addition, we also generated COI data for *P. isimangaliso* specimens collected from four ephemeral vlei localities in the KwaZulu‐Natal province of South Africa. All the latter specimens (*N* = 14) were sequenced for the COI locus using the DNA and sequencing protocol outlined in the current study. The mean haplotype and nucleotide diversity for each of the species was calculated for the COI locus using a hierarchical analysis of molecular variance (AMOVA) performed in Arlequin 3.0 (Excoffier et al., 2005).

The data for each species was imported into RStudio and analyzed using a Kruskall‐Wallis to test for significant differences in height coefficient (CH/CL), haplotype diversity and nucleotide diversity between lentic species and lotic species. The graph was further visualized through the use of an interaction plot.

## 3 RESULTS

### 3.1 Phylogeographic analyses of the COI data for both *Potamonautes* species

**FIGURE 2:**
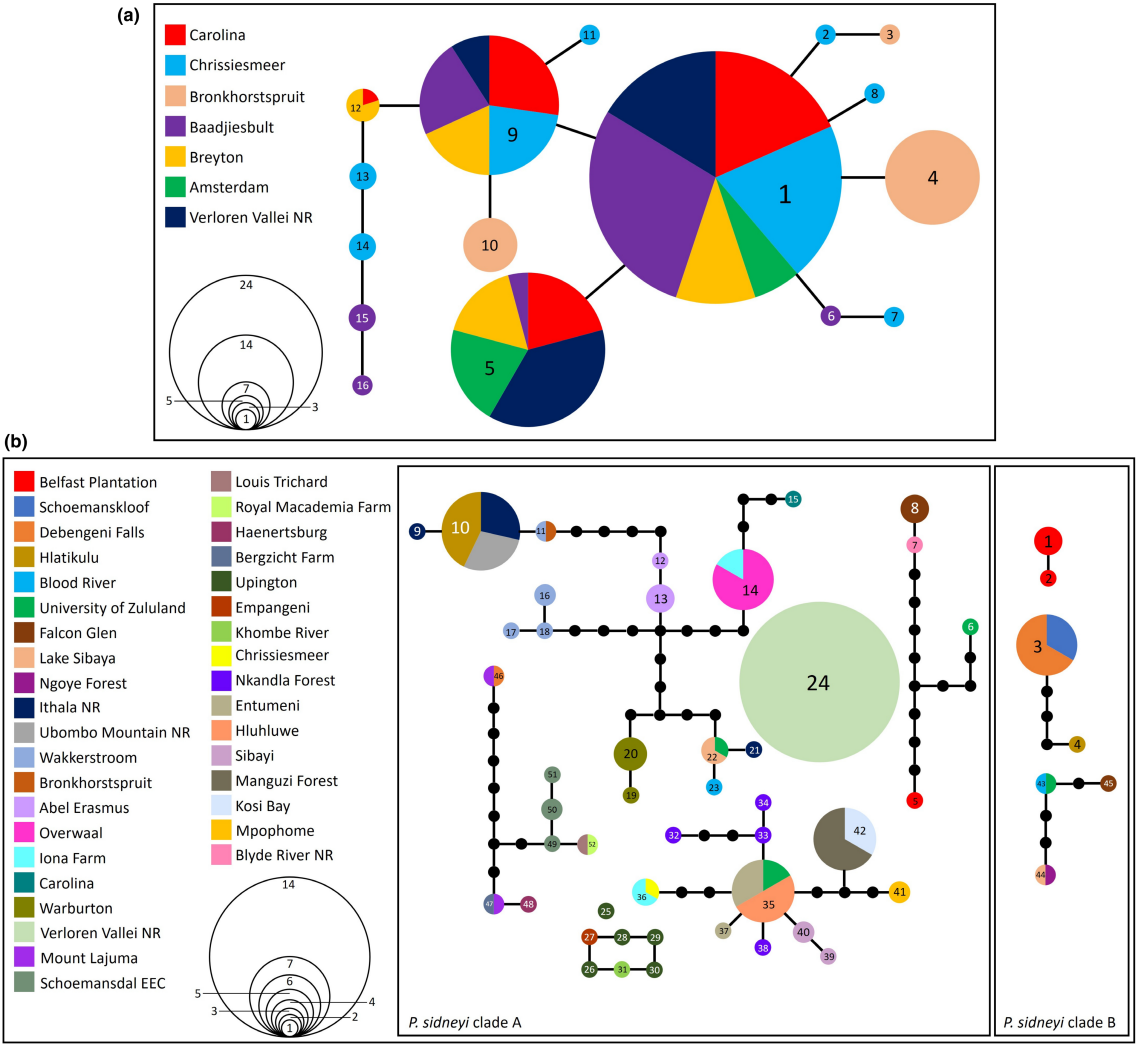
(a) A TCS network for the 16 cytochrome *c* oxidase subunit 1 (COI) haplotypes retrieved for the 138 *Potamonautes flavusjo* specimens sequenced. (b) A TCS network for the 52 cytochrome *c* oxidase subunit 1 (COI) haplotypes retrieved for the 115 *P. sidneyi* specimens sequenced.

**FIGURE 3:**
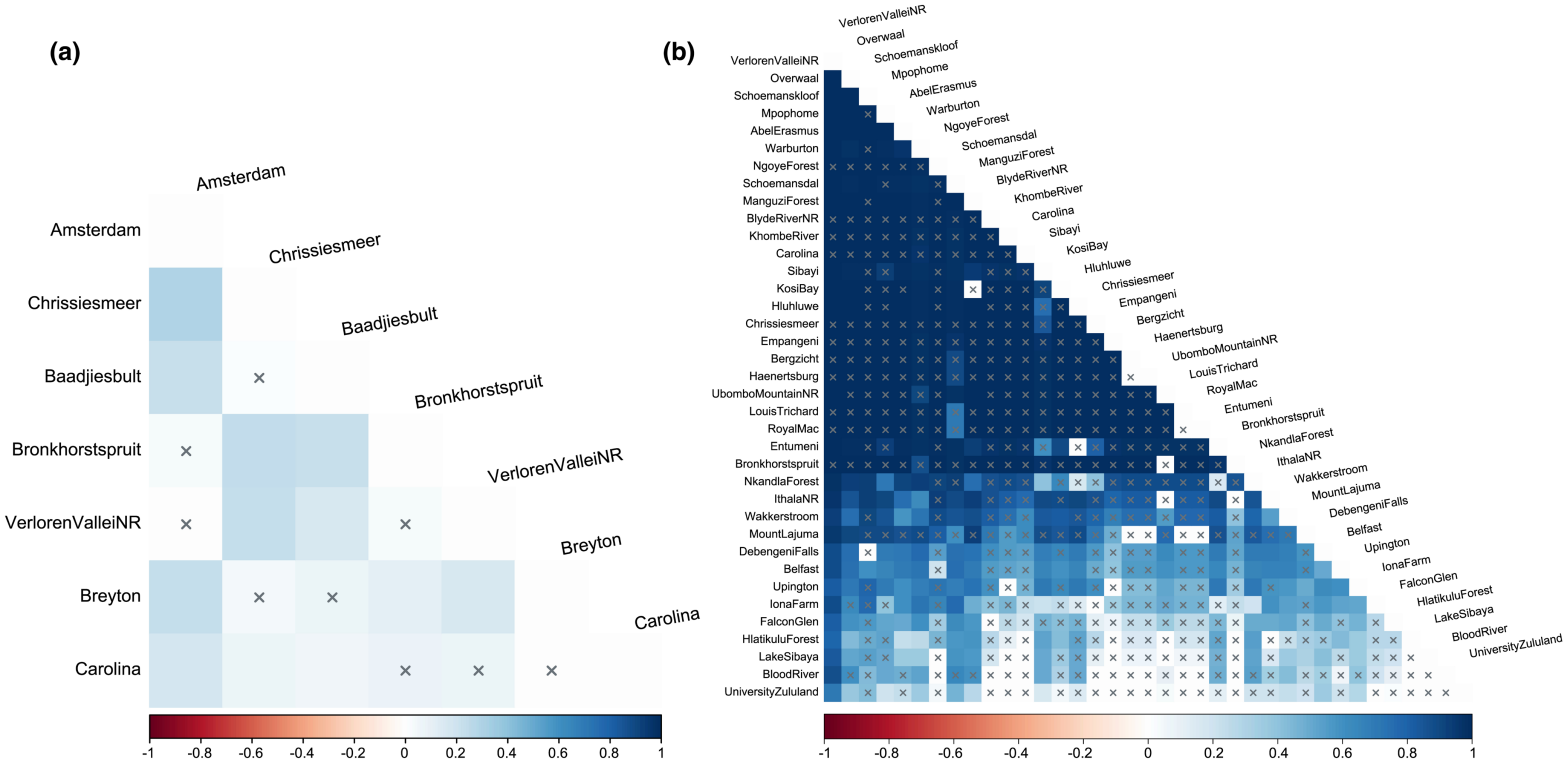
(a) Heatmap diagram with the color showing the strength of the *F*_ST_ value for each respective pairwise *F*_ST_ combination for *Potamonautes flavusjo*. Darker colors show larger levels of *F*_ST_ between different localities, thus being more distinct. The stronger the signal, the more easily the different populations can be told apart from one another by looking at the individuals DNA. The lighter the color, the less distinct. Blocks marked by a superimposed × indicate non‐significance. (b) Heatmap diagram with the color showing the strength of the *F*_ST_ value for each respective pairwise *F*_ST_ combination for *P. sidneyi*. Darker colors show larger levels of *F*_ST_ between different localities, thus being more distinct. The stronger the signal, the more easily the different populations can be told apart from one another by looking at the individuals DNA. The lighter the color, the less distinct. Blocks marked by a superimposed × indicate non‐significance.

A 637 base‐pair (bp) fragment of the COI locus was sequenced for 111 *P. flavusjo* specimens were deposited in GenBank (Accession numbers PP267786–PP267896). The novel COI sequences were combined with the 27 sequences obtained in the previous study (Daniels et al., 2014) to yield a total of 138 sequences. A 95% TCS network collapsed the 138 COI sequences into 16 haplotypes for *P. flavusjo* (Figure 2a) and a single highly interconnected haplocluster was retrieved, with no missing or unsampled haplotypes. Most haplotypes were shared across localities indicating very little variation between localities and high maternal dispersal (Figure 2a). The latter result is corroborated by the AMOVA over all sample localities, that indicated only 12.98% of variation occurred among sample localities (df = 6, SS = 13.49, Va = 0.08, *p* < .001), whereas 87.02% of the variation occurred within populations (df = 131, SS = 75.47, Vb = 0.58, *p* < .001), with an *F*_ST_ of 0.13. The low (−0.040 to 0.300) and statistically significant *F*_ST_ values between sample localities suggest limited genetic differentiation (Figure 3a). Haplotype diversity was high, whereas nucleotide diversity was low (Table 2). Fu's *F*s values were negative for three localities and positive for four sample localities (Table 2). Only one locality, Chrissiesmeer, was statistically significant for this index, thereby limiting our inference (Table 2). Negative Fu's *F*s values are associated with an excess number of haplotypes and recent population expansion, whereas positive values can be associated with a deficiency of haplotypes and can be indicative of a population bottleneck.

**TABLE 2:** List of five population genetic parameters inferred from the cytochrome *c* oxidase subunit 1 (COI) data for the freshwater crab species *Potamonautes flavusjo* and *P. sidneyi*.

Species	Locality name	*N*	Nh	Haplotype diversity (h)	Nucleotide diversity (∏)	Fu's *Fs*	Fu's *Fs p*‐value
*P. flavusjo*	Carolina	21	4	0.7095 ± 0.0543	0.001480 ± 0.001179	−0.09278	.45700
*P. flavusjo*	Chrissiesmeer	25	8	0.7967 ± 0.0610	0.002318 ± 0.001617	−2.77101	.03200
*P. flavusjo*	Bronkhorstspruit	23	3	0.5296 ± 0.0708	0.001799 ± 0.001348	1.70806	.83500
*P. flavusjo*	Baadjiesbult	25	6	0.6533 ± 0.0890	0.002219 ± 0.001565	−0.88902	.27700
*P. flavusjo*	Breyton	17	4	0.7941 ± 0.0393	0.002032 ± 0.001496	0.40398	.59000
*P. flavusjo*	Amsterdam	8	2	0.5357 ± 0.1232	0.000841 ± 0.000881	0.86637	.59500
*P. flavusjo*	Verloren Vallei NR	19	3	0.6199 ± 0.0613	0.001138 ± 0.000992	0.47352	.56600
*P. sidneyi*	Belfast Plantation	5	3	0.7000 ± 0.2184	0.029455 ± 0.018571	4.97380	.97200
*P. sidneyi*	Warburton	5	2	0.4000 ± 0.2373	0.000717 ± 0.000911	0.09021	.31900
*P. sidneyi*	Chrissiesmeer	1	1	1.0000 ± 0.0000	0.000000 ± 0.000000	N/A	N/A
*P. sidneyi*	Iona Farm	3	2	0.6667 ± 0.3143	0.022870 ± 0.017797	4.69033	.94500
*P. sidneyi*	Verloren Vallei NR	14	1	0.0000 ± 0.0000	0.000000 ± 0.000000	N/A	N/A
*P. sidneyi*	Nkandla Forest	4	4	1.0000 ± 0.1768	0.005172 ± 0.004043	−1.23676	.09500
*P. sidneyi*	Ngoye Forest	1	1	1.0000 ± 0.0000	0.000000 ± 0.000000	N/A	N/A
*P. sidneyi*	Lake Sibaya	3	2	0.6667 ± 0.3143	0.042529 ± 0.032461	5.83253	.98400
*P. sidneyi*	Hluhluwe	3	1	0.0000 ± 0.0000	0.000000 ± 0.000000	N/A	N/A
*P. sidneyi*	Mpophome	3	1	0.0000 ± 0.0000	0.000000 ± 0.000000	N/A	N/A
*P. sidneyi*	Entumeni	3	2	0.6667 ± 0.3143	0.001144 ± 0.001426	0.20067	.40600
*P. sidneyi*	Sibayi	3	2	0.6667 ± 0.3143	0.001144 ± 0.001426	0.20067	.39600
*P. sidneyi*	University of Zululand	4	4	1.0000 ± 0.1768	0.049171 ± 0.032812	1.50084	.50400
*P. sidneyi*	Overwaal	5	1	0.0000 ± 0.0000	0.000000 ± 0.000000	N/A	N/A
*P. sidneyi*	Khombe River	1	1	1.0000 ± 0.0000	0.000000 ± 0.000000	N/A	N/A
*P. sidneyi*	Blood River	2	2	1.0000 ± 0.5000	0.063465 ± 0.064317	3.61092	.60600
*P. sidneyi*	Carolina	1	1	1.0000 ± 0.0000	0.000000 ± 0.000000	N/A	N/A
*P. sidneyi*	Falcon Glen	4	2	0.5000 ± 0.2652	0.042024 ± 0.028147	8.03793	.99600
*P. sidneyi*	Abel Erasmus	4	2	0.5000 ± 0.2652	0.000858 ± 0.001063	0.17185	.32900
*P. sidneyi*	Ubombo Mountain NR	2	1	0.0000 ± 0.0000	0.000000 ± 0.000000	N/A	N/A
*P. sidneyi*	Louis Trichard	1	1	1.0000 ± 0.0000	0.000000 ± 0.000000	N/A	N/A
*P. sidneyi*	Royal Macademia Farm	1	1	1.0000 ± 0.0000	0.000000 ± 0.000000	N/A	N/A
*P. sidneyi*	Schoemanskloof	2	1	0.0000 ± 0.0000	0.000000 ± 0.000000	N/A	N/A
*P. sidneyi*	Bergzicht Farm	1	1	1.0000 ± 0.0000	0.000000 ± 0.000000	N/A	N/A
*P. sidneyi*	Haenertsburg	1	1	1.0000 ± 0.0000	0.000000 ± 0.000000	N/A	N/A
*P. sidneyi*	Mount Lajuma	2	2	1.0000 ± 0.5000	0.013722 ± 0.014555	2.07944	.56600
*P. sidneyi*	Debengeni Falls	5	2	0.4000 ± 0.2373	0.026072 ± 0.016480	8.30795	1.00000
*P. sidneyi*	Schoemansdal EEC	4	3	0.8333 ± 0.2224	0.001715 ± 0.001699	−0.88730	.09700
*P. sidneyi*	Blyde River NR	1	1	1.0000 ± 0.0000	0.000000 ± 0.000000	N/A	N/A
*P. sidneyi*	Upington	5	5	1.0000 ± 0.1265	0.019897 ± 0.012737	−0.14456	.29400
*P. sidneyi*	Kosi Bay	2	1	0.0000 ± 0.0000	0.000000 ± 0.000000	N/A	N/A
*P. sidneyi*	Manguzi Forest	4	1	0.0000 ± 0.0000	0.000000 ± 0.000000	N/A	N/A
*P. sidneyi*	Hlatikulu Forest	4	2	0.5000 ± 0.2652	0.033448 ± 0.022547	7.40963	.99400
*P. sidneyi*	Empangeni	1	1	1.0000 ± 0.0000	0.000000 ± 0.000000	N/A	N/A
*P. sidneyi*	Ithala NR	4	3	0.8333 ± 0.2224	0.005146 ± 0.004023	0.73089	.56600
*P. sidneyi*	Wakkerstroom	5	4	0.9000 ± 0.1610	0.007890 ± 0.005440	0.35685	.50800
*P. sidneyi*	Bronkhorstspruit	1	1	1.0000 ± 0.0000	0.000000 ± 0.000000	N/A	N/A

*Note*: *N* represents the number of samples. Nh is the number of haplotypes.

A 583 bp fragment was sequenced for 60 *P. sidneyi* COI sequences and deposited in GenBank (Accession numbers OR430961–OR431010; PP264526–PP264535). The newly generated COI sequence data were combined with 55 COI sequences obtained from five previous studies (Daniels et al., 2014, 2019, 2023; Daniels, Stewart, Gouws, et al., 2002; Gouws et al., 2015) to yield a total of 115 sequences. A 95% TCS network collapsed the 115 COI sequences into 52 haplotypes for *P. sidneyi* (Figure 2b), representing two haploclusters A and B. Haplocluster A, consisted of 45 haplotypes in five connected haploclusers and two unconnected haplotypes. Haplocluster B, consisted of the remaining seven haplotypes arranged in three connected haploclusters. The absence of shared haplotypes both within and between the two main clades (Figure 2a) suggests the lack of maternal dispersal among localities. The latter result was corroborated by the AMOVA analysis over all sample localities. Among all *P. sidneyi* sample localities, 67.78% of the variation occurred among populations (df = 36, SS = 937.35, Va = 7.36, *p* < .001) whilst 32.22% of the variation occurred within populations (df = 78, SS = 273.08, Vb = 3.50, *p* < .001). In haplocluster A, 85.23% of the variation occurred among populations (df = 34, SS = 715.47, Va = 7.150, *p* < .001) whilst 14.77% of the variation occurred within populations (df = 64, SS = 79.28, Vb = 1.23, *p* < .001). In haplocluster B, 98.97% of the variation occurred among populations (df = 8, SS = 138.88, Va = 10.31, *p* < .001) whilst 1.03% of variation occurred within populations (df = 7, SS = 0.75, Vb = 0.10, *p* < .001), with an *F*_ST_ value of 0.678. Pairwise and statistically significant *F*_ST_ values showed marked to moderate levels of genetic differentiation (Figure 3b). The number of samples, haplotypes, polymorphic sites, as well as the amount of haplotype and nucleotide diversity, is reported in Table 2, along with Fu's *F*s (Fu, 1997) values for all 37 localities. The number of haplotypes per locality ranged from one to five, with the highest haplotype and nucleotide diversity found in Blood River (*h* = 1.000 ± 0.5000; *π* = 0.063 ± 0.064), despite only having a sample size of two. The lowest haplotype diversity and nucleotide diversity was in Warburton (*h* = 0.4000 ± 0.237; *π* = 0.0007 ± 0.0009) and Debengeni Falls (*h* = 0.400 ± 0.237; *π* = 0.026 ± 0.016), despite both localities having relatively large sample sizes.

### 3.2 Bayesian skyline analyses for *P. flavusjo* and *P. sidneyi*

**FIGURE 4:**
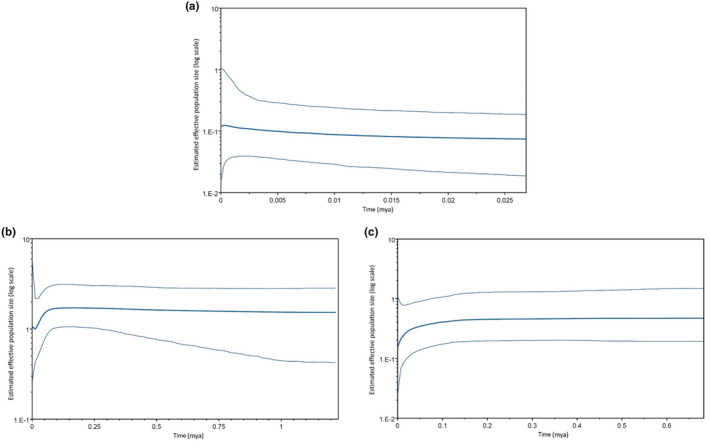
(a) A coalescent Bayesian Skyline analysis output for *Potamonautes flavusjo*. The dark‐blue line is the median estimate of the estimated effective population size. The two light‐blue lines are the upper and lower bounds of the 95% HPD interval. (b) A coalescent Bayesian Skyline analysis output for clade A of the *P. sidneyi* species complex. The dark‐blue line is the median estimate of the estimated effective population size. The two light‐blue lines are the upper and lower bounds of the 95% HPD interval. (c) A coalescent Bayesian Skyline analysis output for clade B of the *P. sidneyi* species complex. The dark‐blue line is the median estimate of the estimated effective population size. The two light‐blue lines are the upper and lower bounds of the 95% HPD interval.

The Bayesian skyline plot for *P. flavusjo* (Figure 4a) indicates a stable population throughout time and suggests that it experiences minimal evolutionary selection pressures. The Bayesian skyline plot for clade A of *P. sidneyi* (Figure 4b) indicates a population that fluctuated throughout time and suggests a population that experiences an overall effective evolutionary selection pressure. The Bayesian skyline plot for clade B of *P. sidneyi* (Figure 4c) suggest a much more stable population size when compared to clade A. This is possibly due to not having enough samples of clade B to accurately make a prediction on how the population fluctuated throughout time.

### 3.3 Phylogenetic analyses, divergence time estimation and species delimitation methods for *P. sidneyi* for the combined COI and 16S rRNA loci

**FIGURE 5:**
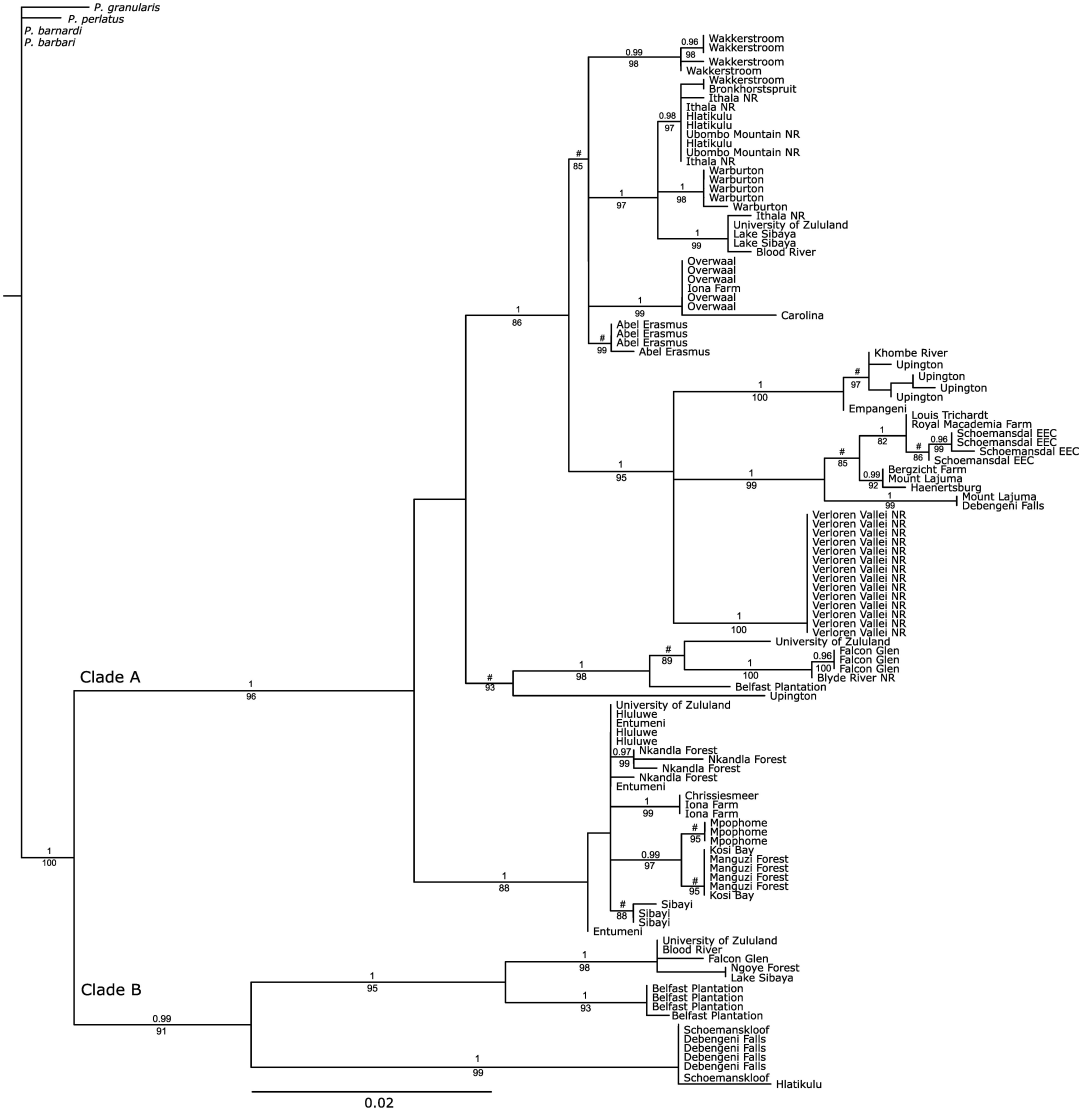
A maximum likelihood phylogenetic tree topology derived from the COI sequence data indicating the evolutionary relationship between the two clades (clades A and B) present in the *Potamonautes sidneyi* species complex. Statistical support for the nodes is provided as posterior probability (p*P* > .95) values above the nodes and bootstrap values below the nodes (p*P* > 75%). A # denotes nodal relationships that were not supported (p*P* < .95) for posterior probability.

The COI data used in the phylogeographic was reduced, a DNA substitution model recalculated and combined with the 16S rRNA data (model not shown). For the 16S rRNA locus, a 432 bp fragment was amplified for sympatric localities and the novel 16S rRNA sequences were deposited in GenBank (Accession numbers: OR430855–OR430870). The DNA substitution model selected using the AIC criteria for 16S rRNA was TIM2 + I (−InL = 875.24). The base frequencies for the 16S rRNA locus was, *A* = 0.365%, *C* = 0.088%, *G* = 0.167%, and *T* = 0.379%, and the rate matrix included *R*(a) [*A*−*C*] = 0.347, *R*(b) [*A*−*G*] = 5.864, *R*(c) [*A*−*T*] = 0.347, *R*(d) [*C*−*G*] = 1.000, *R*(f) [*G*–*T*] =, *R*(e) [*C*−*T*] = 1.270. The BI and ML topologies resulted in near‐identical topologies hence only the ML tree is shown (Figure 5). The ML topology retrieved *P. sidneyi* as monophyletic and revealed two divergent statistically well‐supported clades (A and B). At six localities, the University of Zululand, Blood River, Falcon Glen, Belfast Plantation, Debengeni Falls and Hlatikulu specimens of both clades A and B were present. The uncorrected COI *p*‐distance between the clades A and B was 8.23%.

**FIGURE 6:**
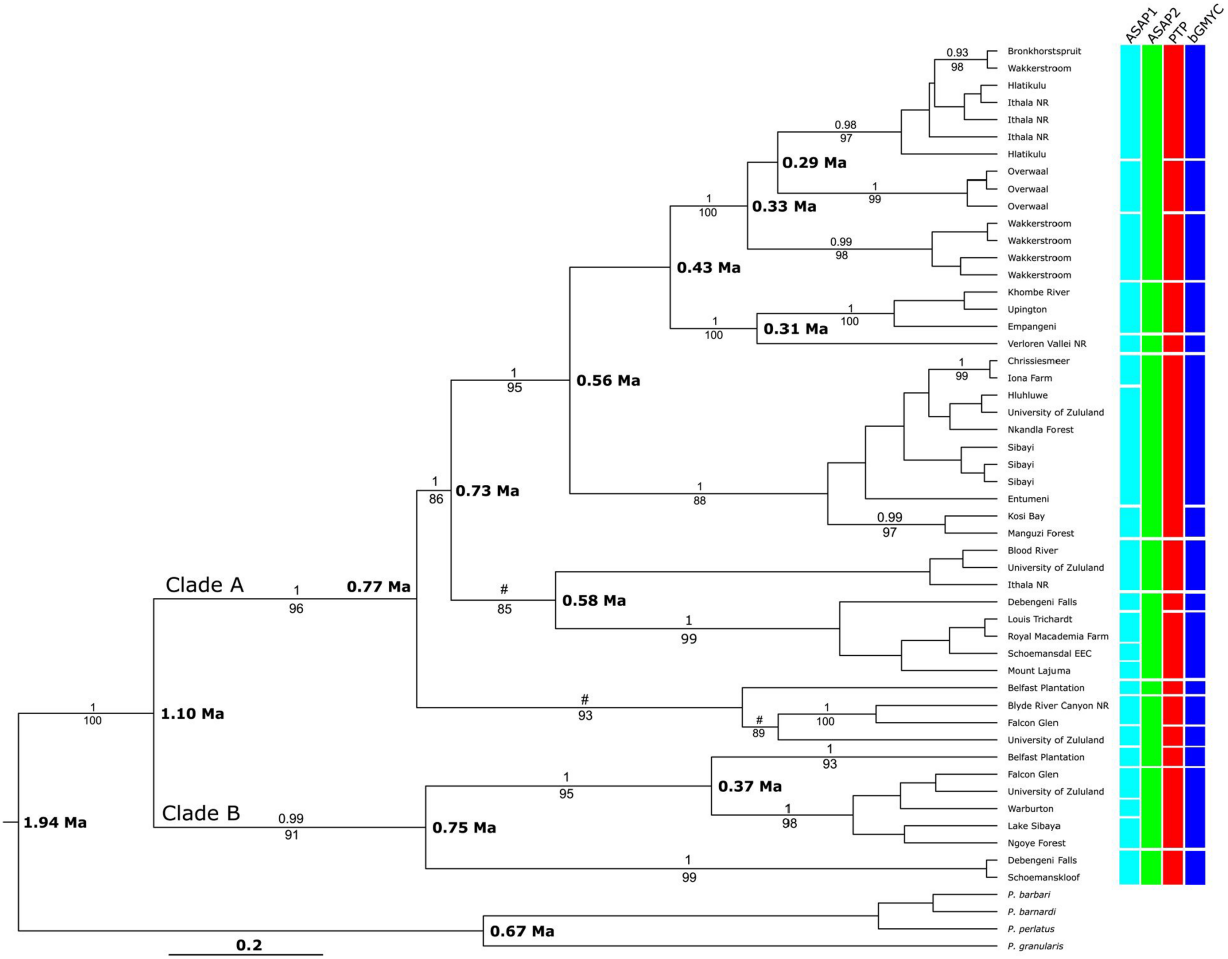
Bayesian phylogenetic tree topology, derived from the COI locus, demonstrating the evolutionary relationships within the *Potamonautes sidneyi* species complex. The three species delimitation methods (ASAP, PTP and bGMYC) together with the combined analyses revealed two clades present within the *P. sidneyi* species complex. The color bars display the results of the delimitation methods where the lines in the matrix separate putative species boundaries based on the observed clusters. Statistical support for nodes for the phylogeny is provided as posterior probability (p*P* > .95) above the nodes and bootstrap values below the node (>75%). A # denotes nodal relationships that were not supported (p*P* < .95) for posterior probability.

The divergence time estimation suggests that *P. sidneyi* originated 1.94 million years ago [95% highest posterior density (HPD) 1.34–2.41 mya]. The divergence between clades A and B is estimated to have occurred 1.10 mya [95% HPD: 0.84–1.44 mya]. The three species delimitation methods over splitting lineages (Figure 6). The ASAP analysis retrieved 21 putative species in the first partition and nine putative species in the second partition (*p* < .01). The PTP analysis recovered 14 putative species. Finally, the bGMYC method retrieved 16 putative species while being highly congruent with the PTP analysis.

### 3.4 Morphometric analyses

**FIGURE 7:**
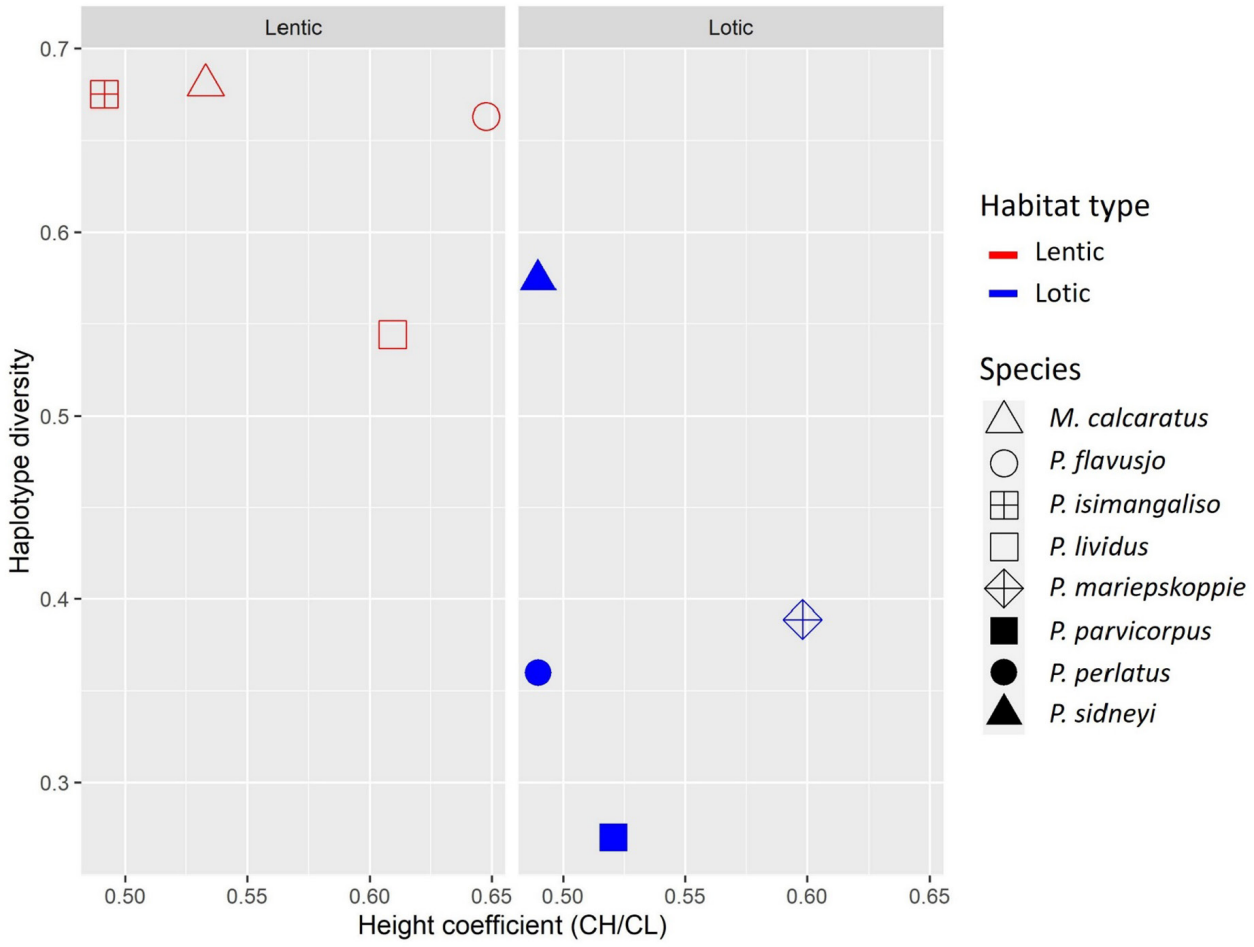
Interaction plot visualizing the relationship between mean haplotype diversity and mean height coefficient of mainland southern African freshwater crab species. The species are divided into two habitat types namely: Lentic (Red) and lotic (Blue).

The list of freshwater crab species of mainland southern Africa, categorized by habitat type, mean height coefficient (CH/CL), mean haplotype diversity and mean nucleotide diversity derived from COI data, are detailed in Table 3. The Kruskal‐Wallis test revealed there were no significant differences between the mean height coefficient of lenticular species compared to lotic species (*p* > .05). Similarly, the Kruskal‐Wallis test revealed that there were no significant differences between the mean nucleotide diversity of lenticular species compared to lotic species (*p* > .05). The Kruskal‐Wallis test demonstrated a notable statistical discrepancy: the mean haplotype diversity among lenticular species significantly surpassed that observed within lotic species (*χ* ^2^ =4.08; df = 1; *p* < .05). These results are displayed in an interaction plot showing the relationship between a species' height coefficient and its haplotype diversity, the lentic species are indicated in red and the lotic species are indicated in blue (Figure 7).

**TABLE 3:** List of mainland southern African freshwater crab species defined by habitat type, mean height coefficient (CH/CL), mean haplotype diversity and mean nucleotide diversity of each species respectively derived from COI sequence data. Where specimens were incorporated from previous studies, these are listed.

Species	Habitat type	Mean height coefficient (CH/CL)	Mean haplotype diversity	Mean nucleotide diversity	Reference study
*M. calcaratus*	Lentic	0.53	0.680	0.00390	Daniels, Stewart, and Cook (2002), Daniels, Stewart, Gouws, et al. (2002)
*P. flavusjo*	Lentic	0.65	0.663	0.00170	Daniels et al. (2014); Present study
*P. isimangaliso*	Lentic	0.49	0.675	0.00170	Present study
*P. lividus*	Lentic	0.61	0.544	0.00220	Daniels et al. (2020)
*P. mariepskoppie*	Lotic	0.60	0.389	0.00160	Unpublished data
*P. parvicorpus*	Lotic	0.52	0.270	0.00056	Wood and Daniels (2016)
*P. perlatus*	Lotic	0.49	0.360	0.00240	Phiri and Daniels (2014)
*P. sidneyi*	Lotic	0.49	0.574	0.01040	Daniels, Stewart, and Cook (2002), Daniels, Stewart, Gouws, et al. (2002), Daniels et al. (2014, 2019, 2023), Gouws et al. (2015); Present study

## 4 DISCUSSION

Contrasting patterns of genetic differentiation were observed between the two freshwater crab species in the present study. In *P. flavusjo*, a wetland specialist, characterized by a highly vaulted carapace, limited genetic differentiation was observed and widespread sharing of haplotypes was present suggesting marked dispersal. Contrastingly, in the habitat generalist *P. sidneyi*, a predominantly riverine species, characterized by a flatter carapace, marked genetic differentiation was observed, evident from the two genetically distinct clades that diverged during the Plio/Pleistocene. Our comprehensive comparative analysis between lentic and lotic freshwater crabs in relation to their carapace morphology, demonstrated no discernible impact on the genetic parameters. Rather, our findings indicate that lentic species demonstrate a discernibly greater aptitude for dispersal, as evidenced from their significant haplotype diversity in comparison to lotic species.

Despite the assumed isolation of the wetland habitats in which *P. flavusjo* occurs (Grobler pers. obs.), haplotypes were frequently shared among localities. A result supporting widespread maternal gene flow was corroborated by the *F*_ST_ analyses (Figures 2a and 3a). For example, haplotypes were shared between Verloren Vallei NR and Chrissiesmeer that are 128 km apart (Figures 1a and 2a). The absence of discernible geographical structuring indicates that the populations of *P. flavusjo* within these wetlands possess high dispersal capabilities. Flooding events would further promote genetic invariance and reduce geographic signatures among semi terrestrial freshwater crabs (Daniels, Stewart, & Cook, 2002; Daniels, Stewart, Gouws, et al., 2002). This could partially explain the shared haplotypes between distant localities like Chrissiesmeer and Verloren Vallei NR, despite their 128 km separation and a 350 m difference in elevation. Alternatively, this haplotype might represent an ancestral retained polymorphism. Comparatively, two other burrowing semi‐terrestrial freshwater crabs have been studied genetically using COI sequence data in the southern African region. *Maritimonautes calcaratus* occurs in natural ephemeral pans in the Kruger National Park and a fine‐scale study revealed moderate genetic structuring using allozyme and COI sequence data (Daniels, Stewart, & Cook, 2002; Daniels, Stewart, Gouws, et al., 2002). In addition, among the Indian Ocean Coastal forest dwelling freshwater crab, *P. lividus*, limited maternal (COI sequence data) and paternal (microsatellite data) gene flow were observed between localities in the Eastern Cape and KwaZulu‐Natal provinces (Daniels et al., 2020). *Potamonautes lividus* has a notably high carapace coefficient (CH/CL = 0.61), suggesting enlarged branchial chambers that would facilitate dispersal over significant distances from water, particularly in conditions of elevated humidity (Daniels et al., 2020). Similarly, the carapace coefficient of *P. flavusjo* is even greater (CH/CL = 0.65), suggesting that it too is adapted for terrestrial mode of life, potentially aiding dispersal.

By contrast, *P. sidneyi* displayed marked genetic differentiating, a result corroborated by the absence of shared haplotypes and by marked *F*_ST_ differentiation. Two main haploclusters were evident based on COI analyses, indicating limited maternal dispersal (Figure 2b). This was supported by the phylogenetic analyses that further supported the two clades (A and B) and the presence of six highly divergent populations where animals occurred in sympatry. The sympatric localities include University of Zululand, Blood River, and Hlatikulu in KwaZulu‐Natal, Falcon Glen and Belfast Plantation in Mpumalanga and Debengeni Falls in the Limpopo province. Cladogenesis in the South African freshwater crabs are closely associated with climatic ameliorations that was initiated at the onset of the Miocene and intensified during the Plio/Pleistocene (Cowling et al., 2009; Daniels et al., 2015, 2023, 2024; Phiri & Daniels, 2014). Due to smaller branchial chambers, it is reasonable to assume that *P. sidneyi* is poorly adapted to xeric conditions and thus cladogenesis was likely promoted during these periods in the Plio/Pleistocene. However, during the Holocene increases in rainfall and frequency of mesic cycles (Scott & Nyakale, 2002) may have allowed for secondary contact after isolation between clades A and B, possibly explaining the contemporary sympatric distributions of these two genetically divergent lineages (S. R. Daniels, personal communication). Divergence time estimation for the *P. sidneyi* species complex suggests a middle Pleistocene origin for the two sympatric clades (Figure 6). The late Miocene is characterized by temperature decline and increased aridification, a pattern also observed in southern Africa (Deacon et al., 1992). This climatic trend persisted and intensified into the Plio/Pleistocene, marked by increased aridification during the late Pliocene and extending into the Pleistocene (de Menocal, 2004; Deacon et al., 1992). Erosion during the Plio/Pleistocene potentially contributed to the formation or elimination of possible migration routes between mountain ranges throughout southern Africa (Partridge et al., 2006). However, stable habitats such as the Soutpansberg in the Limpopo Province of South Africa provided cool and moist conditions and thus refuge for mesic‐adapted or aquatic taxa throughout the aridification process that occurred during the Plio/Pleistocene (Kirchhof et al., 2010). Sea‐level changes during the Plio/Pleistocene altered the coastline and climate of KwaZulu‐Natal (Ramsay, 1996; Wright et al., 2000). In subcontinental southern Africa during the Pleistocene, the persistent and intensified xeric/mesic cycles significantly contributed to heightened habitat fragmentation. This phenomenon, characterized by a general reduction in precipitation and increased temperatures (de Menocal, 2004; Deacon et al., 1992), led to the contraction of inland aquatic habitats towards higher elevations. This habitat restriction likely hindered gene flow among populations in lower‐lying areas, thereby promoting cladogenesis. Consequently, aquatic habitats underwent contraction, with lentic systems particularly impacted, and lotic systems transitioning towards lentic systems due to prevailing water scarcity (Parolin et al., 2008). Moreover, during the Miocene and Pliocene epochs, tectonic uplift facilitated rampant river capture (Deacon et al., 1992). These topographical events significantly altered the landscape of southern Africa and established contemporary rainfall regimes (Deacon et al., 1992; Tyson & Partridge, 2000). Therefore, the tectonic uplift, erosion, climatic improvement, and rearrangement of drainage patterns aforementioned may have led to allopatric divergence. However, if followed by expansion events, the result may be the presence of species occurring in sympatry (Daniels et al., 2024). This Plio/Pleistocene divergence is found in other taxa in southern Africa. For example, the estimated divergence of inland *Amblysomus* lineages (1.8–0.5 million years ago) coincides with Plio/Pleistocene climatic oscillations and strongly suggests that the expansion of more open habitats during drier periods facilitated colonization, with subsequent contraction of these habitats during warmer wetter periods leading to allopatric isolation of populations in patches of suitable mesic habitat (Mynhardt et al., 2015). The divergence of these lineages thus appears to be the result of vicariance associated with expansion and contraction of suitable habitats (Mynhardt et al., 2015). Specifically, in Africa, there were three distinct periods of cooling and aridification around 2.8 ± 0.2, 1.7 ± 0.1, and 1.0 ± 0.2 million years ago, each separated by warmer and more humid intervals (Demenocal, 1995). These changes in climate likely influenced the adaptation, migration, and diversification of ancestral *Amblysomus* taxa through the expansion and contraction of suitable habitats (Mynhardt et al., 2015).

The three species delimitation methods resulted in an oversplitting of lineages with the ASAP method retrieving 21 and nine putative species in the first and second partition respectively. The PTP method recovered 14 putative species and bGMYC retrieved 16 putative species, demonstrating a degree of congruence among them (Daniels et al., 2023). The PTP and bGMYC species delimitation methods exhibited the highest degree of congruence, particularly in cases where oversplitting of lineages occurred, often associated with the presence of divergent specimens and sympatry. The discrepancy observed among the three species delimitation methods is thought to stem from the analyses' sensitivity to differentiate genetic structure and deviations from their underlying assumptions (Daniels et al., 2023; Grobler et al., 2023; Hevin et al., 2022; Sukumaran & Knowles, 2017). For example, the distance‐based ASAP method is considered effective when dealing with low rates of speciation (Puillandre et al., 2021), and both bGMYC and PTP methods address the evolutionary relationships of sequences, with bGMYC optimizing the likelihood score of an ultrametric tree for both intra‐ and interspecific processes, while PTP identifies the transition point from inter‐ to intraspecific processes (Kapli et al., 2017). In our study where marked intraspecific variation and genetic differentiation were observed among populations within the *P. sidneyi* species complex, all three methods overestimated the number of MOTUs, similarly as in the findings reported by Daniels et al. (2023). These species delimitation methods were not useful at discerning the novel species in *P. sidneyi*.

The uncorrected *p*‐distance between *P. sidneyi* specimens from the same locality was 8.23%. This value is typically observed between congeneric freshwater crab species. For example, the uncorrected sequence distance ranging from 7.4% to 10.9% was observed between *P. amathole* and other sister species within its clade, 7.33% between *P. sidneyi* and *P. karooensis*, 10.29% between *P. danielsi* and *P. valles*, 8.65% between *P. mariepskoppie* and *P. ngoyensis*, 10.90% between *P. mariepskoppie* and *P. ntendekaensis* (Daniels et al., 2019, 2021, 2023; Peer et al., 2023). Using the phylogenetic species concept, as outlined by Nixon and Wheeler (1990) our data suggest that there are possibly two species present within *P. sidneyi*. We observed no fixed morphological differences nor any fixed nuclear DNA sequence differences (S. R. Daniels, unpublished data) between the two clades. Consequently, we argue for the use of more sensitive genetic markers such as RADseq to explore evidence of nuclear differentiation in *P. sidneyi* particularly for the six highly divergent sympatric localities where specimens from both clades are present.

Morphologically, *P. sidneyi* displays traits typical of a riverine species, notably featuring a flattened carapace (CH/CL = 0.49), a characteristic also evident in other fluvial species such as *P. perlatus* (CH/CL = 0.49), *P. danielsi* (CH/CL = 0.53) and *P. parvicorpus* (CH/CL = 0.52). *Potamonautes sidneyi* and *P. perlatus* have broad geographical distributions (Daniels et al., 2006, 2023). *Potamonautes danielsi* was found to be sister to *P. sidneyi* species complex and subsequently described as a cryptic species (Peer et al., 2017). Furthermore, within the *P. perlatus* species complex, two novel cryptic species were identified and described: *P. barbarai* and *P. barnardi* (Phiri & Daniels, 2014). This indicates a pronounced inclination towards genetic structuring and subsequent cryptic speciation among riverine freshwater crabs with broad geographical distributions. This pattern is further echoed in the present study study, as two clades are present within the *P. sidneyi* species complex (Figure 5).

Our morphological analyses revealed no statistically significant differences in the carapace height coefficient between lentic and lotic freshwater crab species. This suggests that the height coefficient does not exert a significant influence on the dispersal capability of freshwater crabs and, by extension, their genetic structure. Furthermore, no significant differences were found in nucleotide diversity between lentic and lotic freshwater crab populations. However, the haplotype diversity of lentic freshwater crabs was significantly higher than that of lotic freshwater crabs (Figure 7). This suggests that lentic freshwater crab populations exhibit a higher frequency of shared haplotypes among different localities, implying that these species possess a greater aptitude for dispersal between habitats. In contrast, the habitats of lotic species may hinder dispersal between localities due to barriers (mountains, deserts and dry plains) along the migration routes. Environmental factors such as water flow dynamics, habitat fragmentation, and physical barriers may contribute to the reduced gene flow and higher genetic structure observed in lotic systems. Lotic systems are dynamic systems and are generally associated with more genetic structure when compared with lentic systems in other invertebrate taxa (Drotz et al., 2012; Euclide et al., 2018; Marten et al., 2006). These findings highlight the importance of habitat dynamics in shaping genetic diversity and population connectivity in freshwater crab species, providing valuable insights into their dispersal capabilities and population dynamics.

However, it is important to acknowledge certain limitations in our study. We solely relied on mitochondrial DNA markers for our genetic analyses, omitting nuclear DNA markers. While mitochondrial markers provide valuable insights into maternal lineages and historical demographic patterns, they may lack the resolution offered by nuclear markers, which could reveal finer‐scale genetic structuring and evolutionary relationships (Daniels et al., 2020). Incorporating nuclear markers alongside mitochondrial markers would offer a more comprehensive understanding of genetic diversity and population dynamics within freshwater crab species. Future studies should consider integrating both mitochondrial and nuclear markers to elucidate a more detailed and accurate picture of genetic variation and evolutionary processes in these species. Additionally, our morphological analyses comparing dispersal capabilities between lentic and lotic species were limited by the availability of genetic data. We only had sufficient genetic data for eight species, which may not fully represent the diversity of freshwater crab species in mainland habitats. Expanding the genetic dataset to include more mainland southern African species would enhance the power and robustness of our analysis, enabling more comprehensive conclusions regarding the influence of morphology on dispersal capabilities in freshwater crabs.

In summary, our study illuminates divergent genetic patterns within the freshwater crab species *P. flavusjo* and *P. sidneyi*. *Potamonautes flavusjo*, a wetland habitat specialist, displays limited genetic differentiation and extensive sharing of haplotypes, indicative of robust dispersal abilities. In contrast, *P. sidneyi*, a habitat generalist, exhibits pronounced genetic differentiation with two distinct clades, likely influenced by climatic factors. Notably, *P. flavusjo*, inhabiting lentic environments, shows lower genetic variation compared to the more genetically diverse *P. sidneyi* in lotic habitats. These observations underscore the pivotal role of dispersal capabilities and habitat in shaping crab population genetics. We can thus accept our first hypothesis. However, the disparity in genetic diversity between the generalist and specialist species underscores important considerations for conservation efforts. Specifically, it prompts inquiries into which species may be more resilient or vulnerable to environmental changes such as habitat loss, flooding events, and climate change. By understanding how these factors affect genetic diversity differently between generalist and specialist species, conservation strategies can be tailored to prioritize the protection of genetic diversity hotspots and the preservation of critical habitats, thereby enhancing the long‐term survival prospects of these freshwater crab populations. Furthermore, our analyses suggest that, despite morphological variations, notably in carapace height, we did not find significant evidence to make a conclusive inference on freshwater crab dispersal capabilities based on our data. Therefore, we reject our second hypothesis.

## AUTHOR CONTRIBUTIONS

**Petrus C. J. Grobler:** Data curation (equal); formal analysis (lead); methodology (equal); software (lead); visualization (lead); writing – original draft (lead). **Savel R. Daniels:** Conceptualization (lead); data curation (equal); funding acquisition (lead); project administration (lead); resources (lead); supervision (lead); visualization (supporting); writing – original draft (supporting); writing – review and editing (lead).

## CONFLICT OF INTEREST STATEMENT

No conflict of interest to declare.

## Data Availability

Mitochondrial deoxyribonucleic acid (DNA) sequences generated during the present study were deposited in GenBank. Accession numbers are provided in the manuscript.
